# Institutional Ethnography of Teeth Whitening Industry: How Professional Power Becomes Commercial Power in Dentistry

**DOI:** 10.1111/cdoe.70116

**Published:** 2026-09-25

**Authors:** Rizwana Lala, Peter G. Robinson, Barry J. Gibson

**Affiliations:** 1 Sheffield Centre for Health & Related Research (SCHARR), School of Medicine & Population Health, University of Sheffield, Sheffield, England; 2 Bristol Dental School, University of Bristol, Bristol, England; 3 School of Clinical Dentistry, University of Sheffield, Sheffield, England

**Keywords:** commercial determinants of health, cosmetic dentistry, dental, feminism, general practice, health inequity, inequality, oral heath, power, tooth bleaching

## Abstract

**Objectives:**

This study aimed to demonstrate how power is exercised, and by whom, to expand the practice of teeth whitening in dentistry and the resultant consequences for the public.

**Methods:**

Institutional ethnography (IE) conducted in England that included 100 h of observations at cosmetic dentistry conferences, training and treatments; five field-site and eight in-depth interviews with dentists; 16 in-depth and 3 diary interviews with patients/public. IE analysis identified discourses recurring across field sites and interviews, traced the institutional texts they implied, mapped how institutions activated these texts and generated further texts (work–text–work sequences), then examined disjunctures between institutional discourses and the lived experiences of patients/public.

**Results:**

Two recurring discourses were identified: teeth whitening by beauticians is a “crime” and the “practice of dentistry”, both centred on patient safety. Corporates (Ultradent and Optident) and democratic institutions wielded competing texts (EU Cosmetics Directive and Medical Devices Directive) to promote and resist the sale of higher-peroxide teeth whitening gel in the UK, but corporates failed to secure market entry. Dental institutions used scientific authority to frame teeth whitening as a patient safety issue, opening European markets to higher-concentration products, with dentists as gatekeepers. The Dentists Act 1984 was wielded to exclude competitors such as beauticians. From the patient/public standpoint, disjunctures revealed lived harms including unaffordable dental care, unsafe healthcare spaces, concerns around bodily autonomy and democratic accountability.

**Conclusions:**

Dental institutions and scientific discourse are shaped by power relations, with ethical concepts like safety politically mobilised and functioning as transnational commercial power.

## 1 Introduction

Cosmetic dentistry, including teeth whitening is gaining increasing attention in dentistry, media and culture. In dentistry, tooth discolouration is defined as a change in the colour and translucency of a tooth and divided into two overlapping categorises: extrinsic—surface stains that are responsive to mechanical removal (e.g., prophylactic brushing) and intrinsic—caused by changes in the internal structure of a tooth [1]. The aetiology of tooth discolouration ranges from pathological such as amelogenesis imperfecta, iatrogenic, for example, tetracycline staining and everyday ageing [2].

Dentistry aims to treat pathological and iatrogenic conditions; therefore, treatment of tooth discolouration with procedures such as veneers or teeth whitening is part of clinical practice. The term ‘aesthetic dentistry’ is used to describe treatments for diseased and injured teeth whereby aesthetics or beauty forms part of clinical care, for example, white composite restorations of decayed teeth. The term ‘cosmetic dentistry’ is used for treatments undertaken solely for improving appearance, including tooth colour [3, 4]. Therefore, teeth whitening can be healthcare (aesthetic dentistry) or beauty (cosmetic dentistry). In clinical practice, teeth whitening can involve using chemical agents (peroxide) to oxidise molecules in the teeth to make them lighter [2].

UK and American surveys have demonstrated tooth colour as a key public concern [2, 5]. With this there has been increasing demand and provision of teeth whitening [2]. In 2023, the global teeth whitening market was estimated at USD 7.5 billion and projected to reach USD 10.6 billion by 2030 [6]. The market is highly fragmented, comprising a spectrum of products and services from over the counter (OTC) items such as whitening toothpastes, mouthwashes, and strips to treatments delivered in dental practices and beauty salons. This sector encompasses a diverse range of commercial actors ranging from small-scale practitioners like beauticians and dentists to large multi-national corporations such as Colgate Palmolive and GlaxoSmithKline [6–8].

In public health, there is increasing scrutiny of the ‘commercial determinants of health’—ways in which commercial actors, through various systems, shape health and inequity [9]. Cooperate power is embedded within political and institutional systems [10]. The Lancet series on the commercial determinants of health (CDOH) describes how commercial power reshapes political systems and shifts social norms to align with commercial interest. These processes increase corporate power and wealth and perpetuate inequities [9].

In dentistry, the commercial determinants debate has focused on the ‘common risk factor’ (sugar, ultra-processed food, big pharma, tobacco and alcohol) industries in driving both poor general and oral health [11]. Commercial actors unique to dentistry such as dentists, dental practices (ranging from independent businesses to corporates) and global corporates such as dental product manufacturers have largely been over-looked. Consequently, there is little sustained analysis of how commercial determinants operate within dentistry and how upstream corporate power interacts with broader institutional systems.

The commercial nature of dental practice is established, yet the upstream power dynamics through which commercial and political interests shape routine dental practice, drive poor health and reinforce systemic inequities is underexplored [12]. Moreover, the commercial dimensions of public dental services such as the UK’s NHS dental system are largely ignored. UK General Dental Practices are independent contractors of NHS care, operating within a unique ‘mixed economy’ whereby dentists can undertake NHS and private treatments alongside each other, sometimes for the same patient. A patient can opt for an NHS dental examination and private cosmetic teeth whitening and subsequently pay the relevant NHS and private invoices [13]. Therefore, dentists and dental practices, both publicly funded like the NHS and private, operate as commercial actors.

The European Union (EU) and the UK have some of the strictest regulations on teeth whitening in the world. Bleaching products releasing hydrogen peroxide in teeth, hair, skin and nail are regulated under the *EU Cosmetics Directive and Cosmetic Products Enforcement Regulations 2013* and not dental (*Dentists Act 1984*) or healthcare legislations. Cosmetic legislations permit OTC teeth bleaching products such as whitening toothpastes to release no more than 0.1% hydrogen peroxide, and hair, skin and nail products may release higher levels of peroxide at 12%, 4% and 2% respectively [14, 15]. A 2012 amendment to the EU Cosmetics Directive permitted the sale of teeth products releasing higher concentration peroxide (> 0.1%–6.0%) in European markets but restricted their sale to dentists and used only after a clinical examination by a dentist [16]. In Australia, New Zealand and parts of the US, OTC teeth whitening products may release up to 6% hydrogen peroxide. In New Zealand, non-dentists can provide teeth whitening releasing 7%–12% peroxide, double the maximum concentration permitted from European dentists [17].

Despite widely differing policies, there is little research demonstrating that teeth whitening undertaken by non-dental professionals such as beauticians causes greater public harms. Under European cosmetics legislation, dentists are the only healthcare professionals with exclusive purchasing power for higher concentration hydrogen peroxide products. Health professionals specialising in skin, nail and hair conditions, such as dermatologists or podiatrists, do not have comparable privileges [15, 16]. This lack of regulatory consistency across jurisdictions and professions, coupled with the growing multi-billion-dollar teeth whitening market, prompts examination of how dental regulatory frameworks are shaped; specifically, the relative influence of patient safety concerns, alongside commercial and professional interests.

In countries where dentists have a monopoly over teeth whitening, finite healthcare resources divert away from disease management to cosmetic practice, reducing access to care to prevent and treat oral diseases [18]. Shifting social norms around unrealistic tooth whiteness may contribute to increased appearance-related anxieties. This growing concern is reflected in market data. In 2006, over half of the UK population was concerned with their dental appearance [3]. Two decades later, more than three quarters are self-conscious [19]. Despite this growing significance, teeth whitening research in dentistry has largely focused on technical procedures and clinical outcomes such as different treatment modalities, for example, chairside, home-based, laser and their effectiveness [2]. While this has generated a strong evidence base for the biological and procedural aspects of teeth whitening, little attention has been given to its broader social, ethical and regulatory implications. Beyond Nettleton’s seminal study more than three decades ago [20], dentists’ professional power has attracted little scholarly attention. It is not clear how dentists’ and dental corporate power operate in relation to upstream commercial and political interests to shape poor health and inequity. Thus, the power of actors involved in expanding the teeth whitening industry, and the ways they shape broader institutional systems warrants inquiry.

The aim of this study was to demonstrate how power is exercised, and by whom, to expand the practice of teeth whitening in dentistry and the resultant consequences for the public.

### 1.1 Theoretical Framework

Institutional ethnography (IE), was selected as the method of inquiry [21]. Rather than analysing themes or abstract concepts, IE focuses on how institutions organise everyday activity [21]. It is therefore useful in the study of contested concepts, such as teeth whitening, as it examines teeth whitening as a social activity in which the boundaries between health and beauty are socially negotiated [21–23].

IE is grounded in feminist traditions that assert knowledge is socially situated and unevenly experienced in everyday life. From this lens, dominant forms of knowing such as institutional policies and scientific claims, although presented as neutral and objective, obscure the experiences of marginalised people and may reflect prevailing power relations centred on gendered, racialised, able-bodied and heteronormative norms. Feminist scholarship seeks to make both knowledge and power visible and open to critique [21, 24].

IE extends this approach by analysing how upstream power is wielded from a distance by actors to accrue capital [21]. This aligns with Bourdieu’s (1986) forms of capital: economic capital (money, assets), cultural capital (education, high status jobs), social capital (professional networks) and symbolic (social recognition and legitimacy), the latter is often due to the accumulation of the others. Power operates through these inter-related forms of capital. Actors with valued capital(s) have greater social power to influence what is recognised as normal or legitimate. Capital and power are therefore mutually reinforcing [25].

Central to IE, is the analysis of dominant discourses: shared assumptions about what is ‘normal’ and ‘true’ reproduced across time and space, along with the language and concepts used to express them, and the activities they shape (what people do with those ideas) [21]. Dominant discourses are embedded in institutional texts and recur spatially (different places) and temporally (different times). So, texts shape how people think, understand and enact their everyday *work*. Thus, dominant discourses are rooted in text-mediated relations of power—*ruling relations* [26].

*Ruling relations* are segments of social relations that wield power remotely through *texts* (standardised communications), for example, legislations, protocols. Institutional texts are a product of organisational *work*, that coordinate people’s everyday talk and ‘work’. Yet these texts are created far removed from the lived experiences they govern. IE asserts that in advanced capitalist societies everyone participates in *ruling relations* that shapes everyday work [21]. *Work* is defined generously as activity involving time, effort and intention and not confined to paid labour, thus encompasses oft unrecognised and unpaid (disproportionately gendered) efforts such as care work and emotional labour that allow capital accumulation [21, 27].

### 1.2 Research Design

IE is concerned with discovering *how* things are happening [21, 23]. To avoid uncritical reproduction of institutional accounts IE begins from everyday experiences, accessed through interviews or fieldwork, which constitutes *local* data [28]. During local data collection, the researcher listens for dominant discourses found in multiple places and times and pays attention to institutional texts referenced implicitly or explicitly in those discourses. These institutional texts constitute *translocal* data that shape local activity. The analysis traces *how* the dominant discourse has come about through texts by mapping *work-text-work sequences of action*—*IE maps*. IE maps make *ruling relations* visible by tracing the links between observable work in the field and upstream power [26, 28].

In IE, institutions are systems that organise people’s *work* and discourses in ways that reproduce power relations and social inequities. For the analysis, the researcher anchors the inquiry from the position of a defined group of people (*standpoint group*) and examines how dominant discourses map onto their lived experiences [21].

The analysis identifies *disjunctures* between the dominant discourses and the standpoint group’s lived experiences. Disjunctures are interpretated as manifestations of power differentials [21]. Visual and descriptive mapping (IE maps) highlight gaps in institutional processes, while disjunctures reveal mismatches between lived experiences and organisational expectations. Together, these insights can inform improvements in institutional policies and practices [21, 26].

## 2 Methods and Data Collection

The study was conducted over 15 months between May 2018 and August 2019. Field sites for observations of dentistry conferences were selected based on pragmatic considerations. Conference organisers were contacted by R.L. via email and telephone prior to the conference to notify them of R.L.’s role as a researcher observer. Dentists practising cosmetic dentistry across England were recruited by telephone, email, direct contact and Instagram. Participation involved both dentists and patients consenting to observations during treatment consultations and interviews. Members of the public who had had or were considering cosmetic dentistry including teeth whitening were invited to participate in interviews via advertisements in shops, beauty salons and tattoo parlours in Sheffield and Manchester. During observations, R.L. wore a badge identifying herself as a researcher. Written and verbal consent was obtained from dentists and the patients before treatment observations, with separate written consent for interviews.

Data collection followed an iterative, discovery-orientated, two-level (local and translocal) design by R.L. involving ongoing discussions with P.G.R. and B.J.G. Local data collection included: 100 h of ethnographic observations of cosmetic dental conferences (32 h), cosmetic dentistry treatment sessions (60 h), and dentists’ cosmetic dentistry training (8 h); 5 contextual interviews of dentists whilst at field sites, 8 in-depth interviews with dentists, 16 in-depth interviews with patients (people who have had teeth whitening and cosmetic dentistry) and the public (people contemplating whitening and/or cosmetic dentistry), 3 diary interviews of patients and the public. To seek diversity of experiences sampling recruited informants from diverse ages, ethnic, professional and social groups and genders (Table 1).

### 2.1 Data Analysis

During the fieldwork R.L. wrote fieldnotes to record observations which were subsequently written up as thick descriptions [29]. R.L. analysed the thick descriptions and interviews to identify recurring discourses as they emerged in the language and activities of her interactions and the local settings. Subsequently, R.L. traced the texts—translocal data—that implicitly or explicitly informed the local recurring discourses.

Translocal data analysis examined what texts were *doing* in social organisation, including how they were enacted (work) and whether engagement with these texts generated further texts, producing a work-text-work sequence (IE map) [26]. A diagramming application draw.io was used to draw IE maps.

Drawing on the research team’s critical reflective discussions (see Reflexivity section) the public (which includes patients) were selected as the standpoint group. The analysis examined the disjunctures between the dominant discourses (identified in local and translocal data) and the public’s lived experiences as identified from patient and public interview data.

In summary, data analysis identified dominant discourses, using IE mapping to visualise the links between upstream power and the discourses, and highlighting the disjunctures and consequent power imbalances between institutional authority and the public’s lived experiences.

### 2.2 Data Quality and Triangulation

Data were triangulated across ethnographic field notes, interviews and texts. The GDC policy team was sent the analysis to sense check the analysis and findings. They confirmed they did not have accuracy concerns. The study is presented using standards for reporting qualitative research (SRQR) [30].

### 2.3 Reflexivity

R.L. is a dentist and identifies as a Muslim woman of colour. P.G.R. is a dentist and identifies as a white male. B.J.G. is a sociologist and identifies as an Irish white male. At the time of data collection, R.L. was a practising clinical dentist, and all the researchers taught dental public health at a dental school.

The research team recognised their insider positions and reflected on how our social positions, professional class and familiarity with dentistry shaped the research design, data collection, analysis and interpretations. R.L. experienced cosmetic and teeth whitening upselling at her routine dental visits (outsider position). She noted teeth whitening was increasingly discussed in both professional and personal contexts. R.L. was concerned that the demand for teeth whitening, especially for women, was shaped by social pressures. However, professionally she regarded teeth whitening as the practice of dentistry.

The research team made a purposeful decision to ground the study in the interests of the public (dental public health), with continual efforts to challenge our professional privilege and power. Institutional ethnography cautions insider researchers against ‘institutional capture’, whereby researchers’ immersion in institutional discourse limits alternative words, ideas and ways of knowing [21]. To mitigate this, R.L. wrote a reflexive diary, and the research team held ongoing critical reflective discussions.

R.L.’s plural social and insider-outsider positionalities offered both benefits and constraints. Her familiarity with the UK professional dental landscape allowed her to delve deep into the institutional organisation of teeth whitening within the research timeframe. In her diary, R.L. reflected on her plural, fluid identities along with the tensions and contradictions these generated. She described how her identity as a woman of colour shaped her ethnographic experiences, sometimes disrupting professional loyalties and enabling critical distance from institutional capture. She described physically recoiling at instances of sexism and racialised dynamics at field sites, including talk of ‘fixing’ middle-aged women and sexualised rating of women.

## 3 Results

Two inter-related discourses recurred in the data at multiple times and places: ethnographic fieldwork, interviews and institutional texts. Teeth whitening undertaken by beauticians is a ‘crime’ (dominant discourse 1) and teeth whitening is the ‘practice of dentistry’ (dominant discourse 2). Both dominant discourses converged around the concept of safety (Table 2).

### 3.1 Dominant Discourse 1: Crime

The dominant discourse of crime implicitly references two key institutional texts, the *EU Cosmetics Directive* and the *Dentists Act 1984*, which together make teeth whitening by beauticians illegal. The IE analysis mapped *how* these texts were mediated by different actors to illustrate the mechanisms through which upstream power operates. Mapping was undertaken both descriptively and visually for the *EU Cosmetics Directive* and the *Dentists Act 1984*.

#### 3.1.1 EU Cosmetics Directive

On 31st October 2012, the EU Cosmetics Directive was amended to allow an increase in peroxide concentration of teeth whitening products from a maximum of 0.1% (widely available over the counter) to 6.0%, with the caveat that the sale of products releasing between 0.1% and 6.0% be restricted to dentists [16, 32–34]. Mapping focused on tracing the actors and the forms of work they undertook to drive changes in the Directive that led to an increase in the permitted peroxide concentration in teeth whitening products and positioned dentists as the gatekeepers of higher-concentration (0.1%–6.0%) products.

For nearly a decade (1992–2001), two commercial actors (Ultradent and Optident) attempted to introduce Opalescence, a teeth whitening gel containing 3.4% hydrogen peroxide (well above the 0.1% permitted under the EU Cosmetics Directive before the 2012 amendment) into the UK markets. Table 3 shows the work of commercial and state actors, and the competing texts (EU Cosmetics Directive and Medical Devices Directive) they wielded to market and resist the sale of higher concentration teeth whitening gel in the UK [35–37].

After the House of Lords ruling, all national political mechanisms to introduce higher concentration teeth whitening gel into the UK markets were exhausted. The commercial actors (Ultradent and Optident) were defeated by the UK’s democratic processes, as no further avenues remained within the UK. However, European Union member states can request amendments to EU Directives if supported by scientific opinion [37].

**Figure 1:**
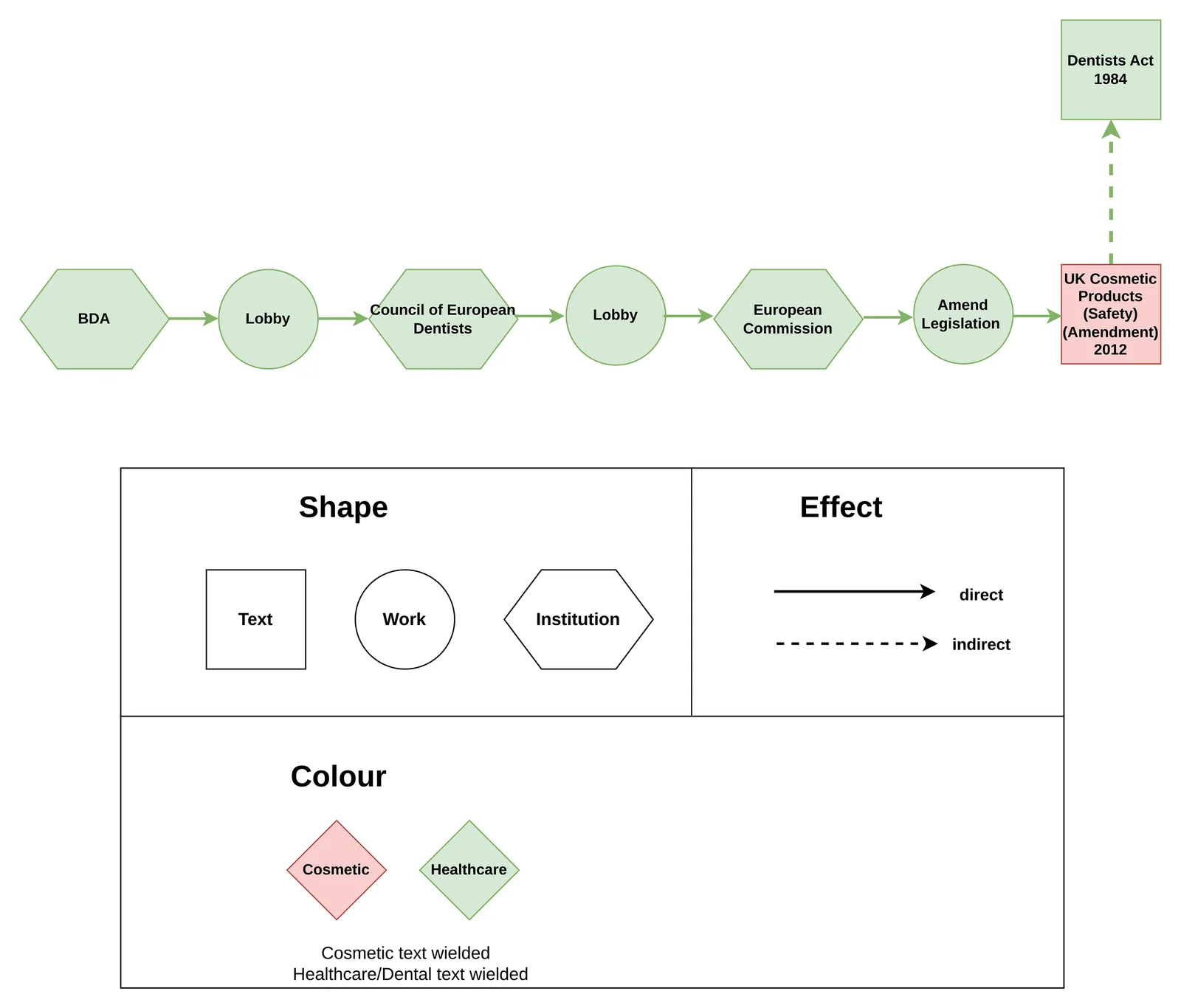
IE map: Institutions working with texts to introduce higher concentration peroxide teeth whitening products into the UK and European markets.

The British Dental Association (BDA), through the Council of European Dentists, undertook work (lobbied) the European Commission “to remove all legal restrictions on dentists so that they can carry out teeth whitening in line with accepted clinical practice” [32]. Dentists used their professional networks (social capital) to influence political spaces and their scientific authority (cultural capital) to shape the discourse on safe teeth whitening. Dentists’ teeth whitening work was identified through analysis of the BDA campaign literature [32]. The concept of safety was wielded to table an amendment to the cosmetics regulations. With the *Cosmetic Product* (*Safety*) (*Amendment*) *2012*, the maximum peroxide concentration of teeth whitening products increased from 0.1% to 6.0%. Due to safety considerations, the sale of teeth whitening products releasing > 0.1%–6.0% peroxide is exclusively restricted to dentists and used only after a clinical examination by a dentist [15] (Figure 1).

Figure 1 shows the work of multiple institutions (BDA, Council of European Dentists, European Commission) that granted dentists exclusive access to higher concentration (> 0.1%–6.0% peroxide) teeth whitening products than those available to the public OTC (< 0.1%). The safety amendment to the EU Cosmetics Directive was ratified into UK law on 31st October 2012 by the Department of Business, Innovation and Skills and not the Department of Health, because this was regarded as cosmetic business and not healthcare [36, 37].

The BDA and Council of European Dentists only represent dentists, and not the wider dental team such as hygienists and therapists. So, it is noteworthy that wider dental professionals, who are regulated by the same professional body as UK dentists (the GDC), were excluded from buying the higher peroxide concentration products. Most significantly, the analysis illustrated how dentists opened the UK and European markets to higher-concentration teeth whitening products, a feat that global commercial actors like Ultradent, despite their presence in more than 130 countries, failed to achieve. Dentists’ scientific authority was mobilised as a multi-national commercial power.

### 3.2 Dominant Discourse 2: The Practice of Dentistry

Teeth whitening was considered ‘the practice of dentistry’ because even advice regarding teeth whitening (irrespective of the peroxide concentration) can only legally be undertaken by dentists as shown by the GDC talk, policy statement and direct quotes from dentists (Table 2).

GDC talk any ‘hand to mouth action’ is dentistry.

It doesn’t matter that it’s not the same bleach, you [non-dentists] still can’t be bleaching patients… according to the GDC, if you sit them there and tell them—breaking the law.

[Francesca, Registered Dentist, 2018]

Products that contain or release less than 0.1% hydrogen peroxide can be legally sold in Europe but that does not mean that unregistered individuals using them to provide tooth whitening treatment makes that treatment legal or safe.

[General Dental Council, Standards and guidance,

2026] [31]

The ‘practice of dentistry’ is not stated in the EU Cosmetics Directive or the EU Medical Devices Directive. Here, the text wielded was the *Dentists Act 1984* (the *Act*).

#### 3.2.1 The Dentists Act 1984 (The Act)

The *Act* outlines the constitution and functions (work) of the General Dental Council (GDC) [38]. The GDC’s remit (work) stated in the *Act* includes ensuring only dental professionals registered with them undertake ‘the practice of dentistry’ in the interest of public safety [38, 39].

**Figure 2:**
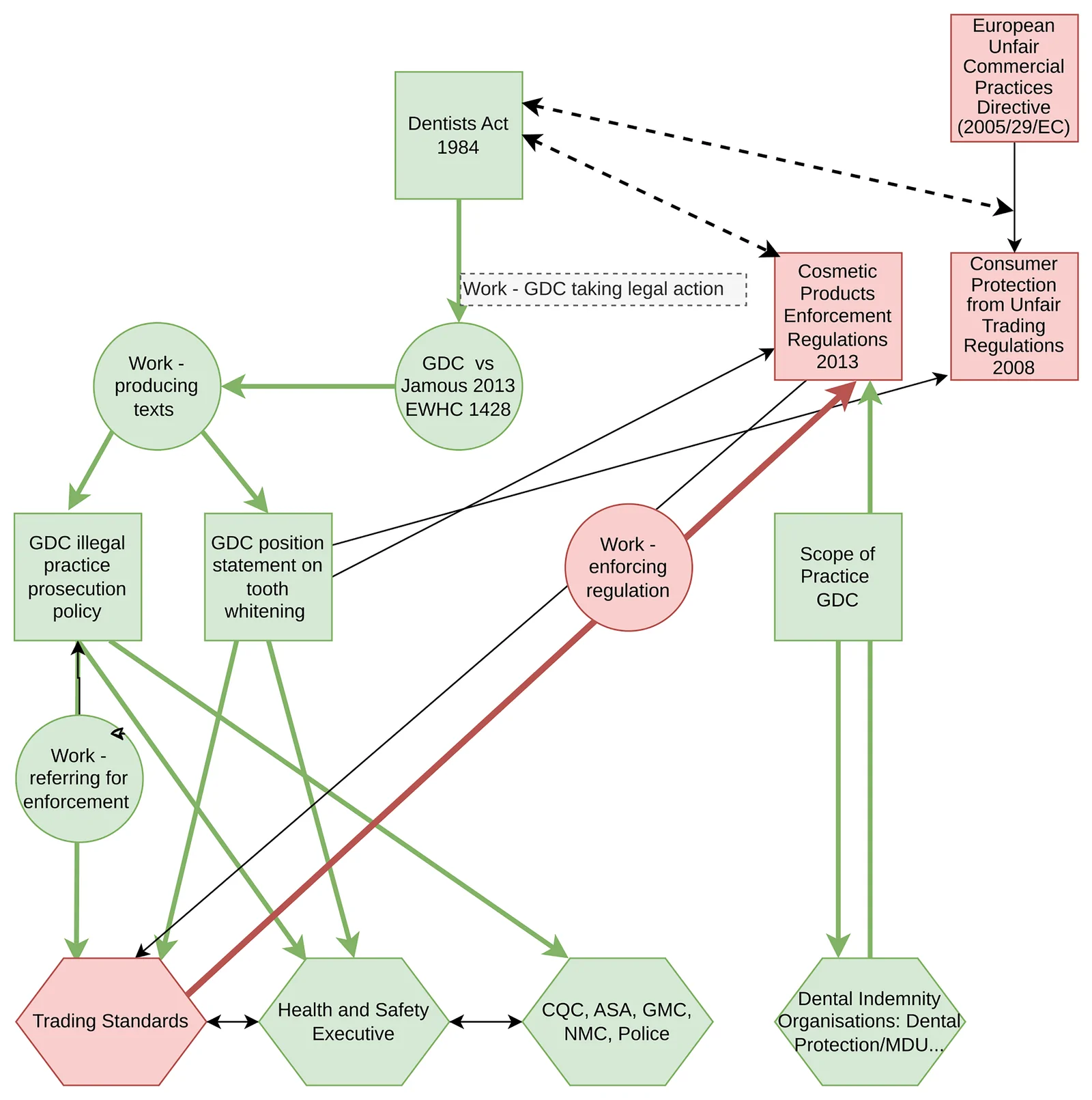
IE map: Work-text-work sequences of action making teeth whitening by non-dental professionals a crime.

Figure 2 shows the work-text-work sequences of action of making teeth whitening by beauticians a crime.

Figure 2 maps how with the authority of the *Act*, the GDC brought a private prosecution (work) against a beautician (Lorna Jamous) for the illegal practice of dentistry. Jamous had undertaken a day-long course on teeth whitening and was indemnified to provide the procedure. She subsequently undertook teeth whitening on 27th December 2011 prior to the safety amendment ratification (31st October 2012) to the EU Cosmetics Directive [40].

Jamous provided teeth whitening in compliance with *all* UK and EU cosmetics laws at the time, only using products containing < 0.1% hydrogen peroxide that were freely available OTC for public use. However, the GDC argued that teeth whitening (irrespective of the peroxide concentration) is the practice of dentistry; thus, Jamous, a non-dental professional, was in breach of the *Act*.

…the practice of dentistry shall be deemed to include the performance of any such operation and the giving of any such treatment, advice or attendance as is usually performed or given by dentists; and any person who performs any operation or gives any treatment, advice or attendance on or to any person as preparatory to or for the purpose of or in connection with the fitting of dentures, artificial teeth or other dental appliances shall be deemed to have practised dentistry within the meaning of this Act.

[Section 37 [1] *The Dentists Act 1984]*

The *Act* does not exhaustively list procedures that constitute the practice of dentistry. Section 37 quoted above shows that the only procedures it details are fitting dentures, artificial teeth and dental appliances. Jamous had not used a dental appliance such as a mouthguard to whiten teeth. Jamous’s defence argued that the products she used were freely available to the public such as whitening toothpaste. Therefore, Jamous’s actions were comparable to parents brushing their children’s teeth and not the practice of dentistry [40, 41].

The prosecution presented an expert witness (a dentist) who described the dangers of teeth whitening, and in doing so mobilised the concept of safety. The prosecution also presented a text, the GDC’s *Scope of Practice* (SOP) document as evidence that teeth whitening is the practice of dentistry [40–42].

**Figure 3:**
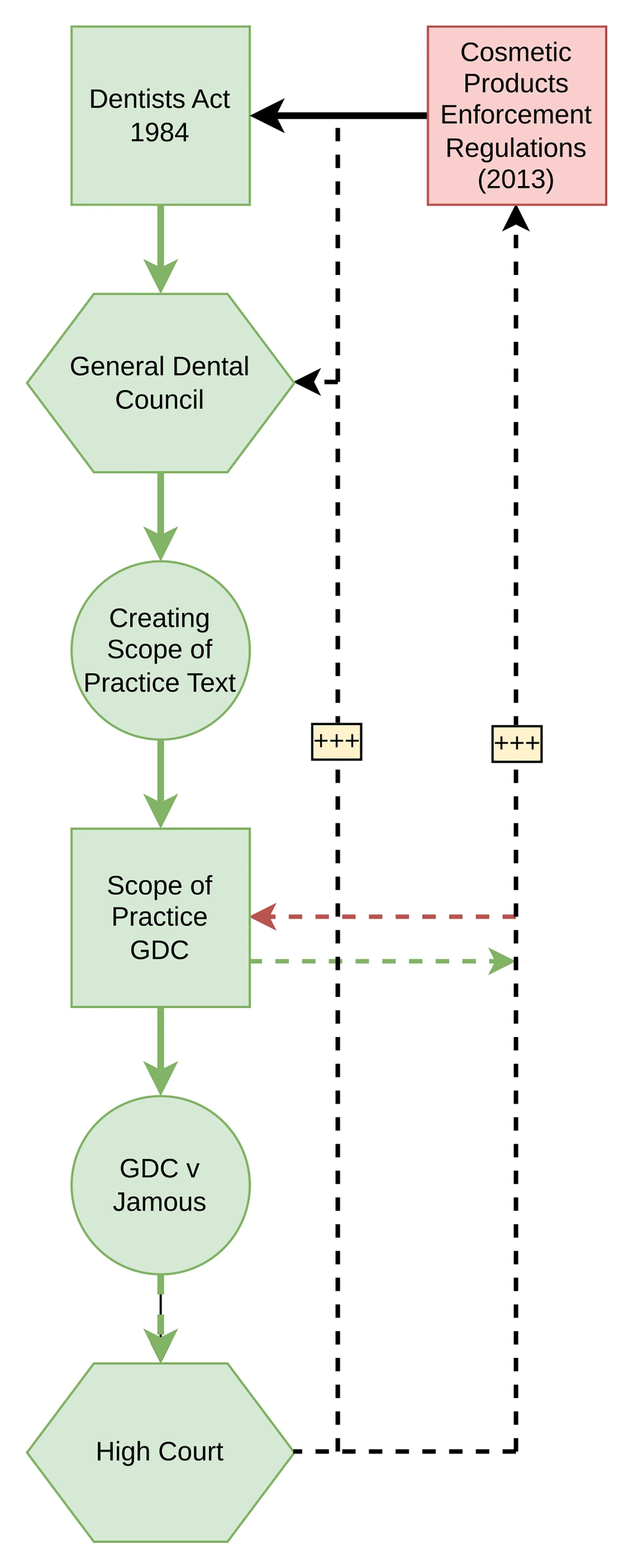
IE map: Work-text-work sequences of action making the SOP.

Figure 3 shows that the GDC itself produced the SOP because section 26B of the *Act* gives the GDC authority to produce ‘texts’ related to dental professionals*’* scope of practice.

The Council [GDC] shall prepare and from time to time issue guidance as to the standards of conduct, performance and practice expected of registered dentists.

[Section 26B [1]: the Dentists Act 1984].

The GDC comprises lay people and registered dental professionals and dentists and has a statutory requirement (outlined in the *Act*) to include dental professionals in the production of the SOP. Therefore, dentists themselves were involved in producing the SOP. After producing the SOP, the GDC used the text to legitimate its further work of taking legal action against Jamous. But the SOP also failed to list all treatments that constitute the practice of dentistry. The only references to teeth whitening were those stating that hygienists, therapists and clinical dental technicians could undertake teeth whitening to the prescription of a dentist [42]. Thus, the District Judge ruled that the purpose of the SOP was to regulate the profession rather than identify what constitutes the practice of dentistry. The GDC was defeated.

The GDC appealed the District Judge’s ruling in the High Court arguing that teeth whitening “is usually performed by dentists” [40, 41] so was the practice of dentistry. The High Court Judge ruled that whilst the District Judge’s assessment was true, the SOP presented some evidence that teeth whitening is usually done by dentists. Despite this judgement, the High Court Judge articulated that the mere mention of an activity in the SOP is not sufficient evidence that it is the practice of dentistry. The GDC could not just include procedures in the SOP and classify them as the practice of dentistry. Nevertheless, the High Court concluded that teeth whitening (irrespective of the type/concentration of product used) was the practice of dentistry in accordance with section 37 of the *Act*. Therefore, Jamous was in breach of section 38 of the *Act* which makes the practice of dentistry by non-registered professionals a criminal offence. Sections 40 and 41 of the *Act* also make it illegal for personnel not registered with the GDC to undertake “the business of dentistry”, that is, receive payments for dentistry making Jamous also guilty of these crimes as she was duly paid for her services [38, 40, 41].

On 16th September 2013, Jamous was sentenced to a conditional discharge for 12 months and ordered to pay £350 in costs. In August 2014, Jamous applied to the Southwark Crown Court to appeal the High Court decision, but the application was refused. Subsequently, without legal representation, she pursued a judicial review of the Crown Court decision which was also refused. Lorna Jamous was ordered to pay £1500 towards the GDC costs [43]. The High Court ruling still stands. Teeth whitening (irrespective of the products used) is the practice of dentistry. With this legal legitimacy, the GDC undertook more work to create more texts: the GDC positioning statement on tooth whitening and the GDC illegal practice prosecution policy (IPPP) [44, 45] as mapped by Figure 2. Figure 2 also illustrates how these GDC texts state the work of more institutions: Health and Safety Executive, Care Quality Commission (CQC), Advertising Standards Agency (ASA), General Medical Council (GMC), Nursing and Midwifery Council (NMC) and the police to stop any illegal practice of teeth whitening [44, 45]. Thus, multiple institutions and texts create the recurring knowledge or ideological dominant discourse (across multiple sites and times) that teeth whitening by beauticians is a crime.

Figure 2 illustrates that despite the GDC producing texts (*positioning statement on tooth whitening* and the *IPPP*) to enforce the *Dentists Act 1984*, those texts principally authorise Trading Standards to enforce the *Cosmetic Products Enforcement Regulations 2013* and not the *Dentists Act 1984* due to the absence of teeth whitening from the *Act* (*Dentists Act, 1984*) [44, 45]. Teeth whitening is governed by *cosmetics legislations* and not healthcare or dental regulations. Breach of cosmetics regulations results in prosecutions under consumer protection laws, *Consumer Protection from Unfair Trading Regulations* (*2008*) [46].

Local and translocal data confirmed how the governance of teeth whitening remains within cosmetic and not dental legislations demonstrated by the quotes below:

Yes, I used to think it was impressive that the GDC had made sure that it was… it was one of the few things the GDC did that I thought was good, that they made sure that tooth whitening, for example, was the business of dentistry. And then I found out that they had no jurisdiction to do anything about it anyway.

(Paul, GDC registered dentist, 2019).

The GDC does not bring criminal prosecutions of breaches of the regulations [(Cosmetic)] as this role is undertaken by Trading Standards.

[Focus on Standards, The 9 Principles, General Dental

Council, 2013] [47].

The data and IE maps (Figures 1–3) reveal an institutional discursive conflict; teeth whitening as the practice of dentistry or cosmetic practice. Teeth whitening (in 2026) is absent from the *Act* and sits under cosmetics regulations, and therefore, regarded a beauty practice, and it was argued as such by democratic institutions (Table 3). The discursive conflict has arisen due to multiple actors with conflicting interests; those who operated to enter and control the teeth whitening market, and those attempting to uphold democratic processes and public protections. Table 4 maps the institutional actors that worked to open or resist the UK and European teeth whitening markets, tracing their divergent interests and forms of accountability.

Ultimately, the GDC, alongside wider institutions, came to define teeth whitening as ‘the practice of dentistry’ explicitly referencing the Dentists Act 1984. This reference across multiple institutions makes teeth whitening by beauticians a crime and transforms a beauty practice (still governed under cosmetic regulations) into healthcare (the practice of dentistry). This transformation creates several disjunctures which were identified through further analysis of local and translocal (Dentists Act 1984) data.

### 3.3 Disjunctures From the Standpoint of the Public

#### 3.3.1 Bodily Autonomy

The GDC v. Jamous case law has legislated who can touch people’s bodies and even speak about aspects of the body (mouth), raising concerns around bodily autonomy. If people exercise their agency to pursue traditional options for teeth whitening from non-dental professionals, they are institutionally labelled as victims of crime.

#### 3.3.2 Availability and Affordability Dental Care

Across Europe, teeth whitening can now only be accessed via elite, professionalised spaces. This disproportionately impacts minoritised and economically disadvantaged communities who struggle to access exclusive medical care. Dentists’ monopoly, on both access to teeth whitening products and as providers of advice and treatment, leads to concerns about access and affordability of care as seen by the direct quote:

“I think it’s very expensive for what you’re getting done, compared to your NHS costs. So, it wasn’t until I moved in with my partner, that it could have become anything near affordable for me…I took some money out of my savings.”

[Charlene 2019: Cosmetic Dentistry Patient].

NHS space and resources (workforce) are diverted towards elective private cosmetic care. My informants were aware of this disjuncture as noted by Avatel in her diary as she contemplated having teeth whitening:

“I feel less certain today [about having teeth whitening] because of two chance encounters, one with the young woman who sells the Big Issue… She is only 16 but has had a tooth removed recently and is in a lot of pain, and told me so. It made me think that if I’m prepared to spend several hundred pounds on non-essential, potentially damaging treatment on my teeth, would it not be better spent on her, given that she actually needs it?!…”

[Avatel: Member of the Public, Diary, 2019].

#### 3.3.3 Narrowing Body Ideals

The opening of markets to teeth whitening has led to greater uptake of whitening and cosmetic dentistry [6, 7] which is narrowing dental beauty norms as seen by the quotes below.

“I think there should be more emphasis on the fact that…you don’t have to look like the piano keyboard or whatever, to be acceptable. I think there’s a lot of that. But I don’t know how that could be promoted. It wouldn’t be promoted. It doesn’t sell anything. It’s about selling, isn’t it?”

[Janice, Member of the Public, 2019].

“The bright white, ridiculous smile…just looks so normal to me now”

[Tamara: Cosmetic Dentistry Patient, Diary, 2018].

The growing discontent people experience with the appearance of their teeth [3, 19] is confirmed by the data:

“…it’s really hard to not just start to kind of see that [the same dental appearance] as that’s what you must look like…”

[Tamara: Cosmetic Dentistry Patient, 2018].

The data showed narrowing beauty norms around teeth whitening are experienced differentially across social categories. Tamara reflects on this in her diary:

“ …the difference between how men and women feel about their teeth: for women it’s a sign of beauty and important that that they are white…but for men their teeth are functional and it’s about them staying healthy…”

[Tamara: Cosmetic Dentistry Patient, Diary, 2018].

#### 3.3.4 Unsafe Healthcare Spaces (Physical and Digital)

During the ethnographic observations R.L. noted images of straight white teeth and teeth whitening promotions in all field sites: conferences and dental practices (NHS and private). In the mixed economy of dental practices, clinical spaces (both publicly funded like the NHS and private) are used to promote narrow body ideals as noted by Avatel’s diary:

“On the front door of the dentist (NHS), next to the charity shops and the Greggs, was an A4 poster with a smiling tooth emoji announcing a whole tooth whitening course for £350”

[Avatel: Member of the Public, Diary, 2019].

Despite this, teeth whitening is not available on the NHS as it is considered cosmetic and not healthcare. Nonetheless, whiter teeth are becoming the social norm; therefore, people feel there should be greater affordable availability as articulated by Elsie:

“…not everyone can afford to come private, I mean it’s expensive. I do think it should be more readily available for people at a more affordable cost for people.”

[Elsie: Cosmetic Dentistry Patient, 2019]

Fieldnotes and interview data demonstrated that dentists used social media, especially visual platforms like Instagram, to promote idealised white smiles, often through before and after images showcasing treatment outcomes, to expand business revenue as seen by the quote by Zahra:

“…but I noticed the impact of it [Instagram]. I did notice that patients who came in said, “I’ve seen your photos,” and then I think, subconsciously, when I realised that it actually had that power… I didn’t intend it to become a big source, but it has been. I think it definitely has been a help, from a business point of view”

[Zahra: Registered Dentist, 2019].

#### 3.3.5 Financial Stress

Promotions of idealised white smiles, narrowing norms and the limited affordability of private cosmetic dentistry can create social and financial pressures.

During the fieldwork and interviews, R.L. observed mechanisms that facilitated access to finance and debt for dental treatments. Examples include stands for Snowbird Finance at the British Dental Conference and Dentistry Shows, and payment options offered through dental practices acting as credit brokers, as seen in Joe’s quote:

“Then we have patients who really, really require it [teeth whitening and cosmetic dentistry] but the NHS isn’t able to help them and so they would take out a finance plan to help them achieve it…we offer that sort of option.”

[Joe: Registered Dentist, 2019]

#### 3.3.6 Institutional Breach of Public Trust

Public safety held a central concern in institutional processes. The GDC’s primary purpose stated in the *Act* is public safety:

“The over-arching objective of the Council [GDC] in exercising their functions under this Act is the protection of the public.”

[Section 1(1ZA) the Dentists Act 1984]

The concept of safety served a discursive link across both dominant discourses; however, it was mobilised in ways that served commercial interests rather than public health.

Dentists (through the Council of European Dentists) were positioned to influence the EU Commission to open European markets to higher concentration teeth whitening products. With this, the Cosmetic Product (*Safety*) (Amendment) 2012 authorised an increase in the permitted peroxide concentration of teeth bleaching products, with safe purchase and handling of these products institutionally attributed to only dentists. The concept of safety was also wielded by dentists (via the expert witness in the GDC v Jamous case) which removed competitor teeth whitening practitioners (beauticians). The quote by Richard demonstrated dentists’ awareness of their competitive advantage in the practice of teeth whitening:

“There is regulation there at the moment in terms of beauticians not being allowed to do treatments on teeth and I think that’s very welcome and I hope it stays there. I’d like to see more prosecutions”

[Richard: Registered Dentist, 2019].

Although dentists are commercial actors, their commercial interests were organised out of the dominant discourses. The absence of explicit talk about profit enabled dentists to gain market monopoly while presenting their position aligned with the language of public safety ethics. This concept of safety was especially effective because of the high level of public trust afforded to scientific authority and healthcare professionals as shown by the quote below:

“I would be much more inclined to go to a dentist rather than a beautician because I don’t think I would trust their training …I would go to a dentist and I’d try to go to as reputable a dentist as possible. And a specialist in tooth whitening, probably.”

[Avatel: Member of the public, 2019]

This exposed breaches of public trust by dental institutions and broader failures in democratic accountability. Moreover, safety was understood in a narrow sense, focusing on physical body harms and broader dimensions of safety such as fostering psychologically safe healthcare spaces were overlooked.

Collectively, disjunctures demonstrated the broad-ranging work demanded of the public by the expanding practice of teeth whitening, encompassing administrative work to navigate dental access, secure finance for treatments and emotional work involved in negotiating healthcare environments saturated with idealised smile images and narrowing body ideals.

## 4 Discussion

Between 1992 and 2001, two multi-national corporates (Ultradent and Optident) mobilised the *EU Cosmetics Directive* and *EU Medical Directive* to open UK and European markets to higher concentration peroxide teeth whitening products, but were unsuccessful due to UK regulatory and democratic processes. In contrast, institutions representing dentists (British Dental Association and European Council of Dentists) successfully mobilised professional networks and scientific authority to frame higher-peroxide concentration teeth whitening products as a clinical and patient safety issue, securing legislative changes that positioned dentists as gatekeepers for higher-peroxide teeth whitening products. In addition, teeth whitening has been institutionally and legally constructed as the practice of dentistry in the UK through interpretation of the *Dentists Act 1984*, making teeth whitening by non-registered dental professionals such as beauticians a criminal offence. Taken together, these findings illustrate how an everyday clinical practice (teeth whitening) is entangled with complex social, institutional and political processes associated with opening and expanding commercial markets with dentists emerging as key actors in these processes, shaping both regulation and market entry.

The disjunctures highlight the broad-ranging administrative, financial and emotional work demanded of the public by the expanding practice of teeth whitening. These include navigating access to affordable dental care, securing treatment finance and negotiating healthcare systems saturated with idealised smile images. These dynamics illustrate the diverse forms of labour extracted from the public to sustain processes of global capital accumulation [21, 27]. Disjunctures also reveal how commercial dynamics shape public experiences of safety, including broader emotional and financial dimensions that are overlooked when safety is narrowly conceptualised solely around clinical risk. Patients and the public in this study identify cost as a barrier to accessing teeth whitening. This aligns with US evidence suggesting a protectionist stance on teeth whitening as seen in the UK and Europe has cost implications for the public [17, 48]. The combination of high-cost private dental teeth whitening, and the highly restricted availability of OTC alternatives may be fuelling the growing circulation of dangerous illegal whitening products (exceeding 6.0% hydrogen peroxide) being sold online [49]. Paradoxically, this challenges the concept of safety that is discursively linked to dominant institutional discourses.

A key limitation of this study is the exclusion of beauticians and patients who received teeth whitening from them, due to ethical complexities associated with researching illegal practice. Moreover, recruitment of informants—dentists, patients and the public—was limited to England. Teeth whitening operates across parallel systems of provision that vary across jurisdictions, including contexts where beauticians can legally operate [17]. However, this study does not capture unregulated and alternatively regulated whitening practices that are part of the same broader system, especially in the context of globalisation and medical tourism.

Nonetheless, this is the first study since Nettleton’s seminal work to undertake a sustained analysis of dentists’ professional power. It echoes her findings that dentists wield power, authority and control through their specialised (scientific) knowledge [20]. Dental ethics (in this study safety) is a negotiated form of professional power that can limit patients’ rights. For teeth whitening, safety is organised into the discourse and dentists’ commercial interests are organised out, which functions to legitimate restrictions on public/patients’ rights such as bodily autonomy and access to safe, affordable healthcare. Silence and elisions emerge as key mechanisms through which discursive power is enacted.

The analysis of professional power addresses an important gap to the CDOH literature. Although the CDOH framework highlights overcommercialisation of healthcare causes health harms, it focuses on private healthcare, and the role of health professionals is limited to moving beyond the biomedical model [9]. However, our findings demonstrate overcommercialisation is also embedded in public healthcare systems like the NHS. Whilst beauty-related concerns would not be regarded as requiring medical intervention if dentists moved beyond the biomedical model to ‘fix’ smiles, our findings demonstrate that health professionals are also shaped by, and involved in, commercial and political processes. This complicates the boundaries between healthcare, commercial activity and political power.

Complications arise because actors have multiple and conflicting interests. The EU Notified Body is a for-profit commercial actor tasked with ensuring patient safety. The Department of Trade and Industry is a democratic institution regulating patient safety but is also concerned with expanding markets. Dentists combine healthcare and commercial activity. These competing interests illustrate that regulation is shaped by political tensions that require negotiation. Serban et al. [50] described the political dimensions of the commercial determinants from the ‘common risk factor’ industries in dentistry. They suggest the resultant oral health inequities may be countered through scientific evidence orientated policy actions [50]. However, our analysis traces empirically how scientific legitimacy itself is mobilised as political and commercial power. In Bourdieusian terms, dentists’ professional networks (social capital), alongside the scientific authority (cultural capital) of medical actors more broadly, can be converted into symbolic capital (scientific and professional legitimacy) which can be mobilised politically in support of transnational commercial interests [51].

Commercial power is not merely market activity; it is produced, negotiated and at times protected politically [52]. The protection of commercial power through political processes is highlighted in GDC v Jamous. The ‘practice of dentistry’ (discourse 2) is a generic or ‘shell’ term in the *Act*. In IE shell terms appear concrete, but they function as the institutional abstraction and neutral façade necessary to hide complex activity happening [21, 53]. Thus, despite the absence of the teeth whitening in the *Act* or the shell term ‘the practice of dentistry’; those with power and symbolic capital (dentists, GDC, High Court) fill the shell term with teeth whitening, protecting dentists’ monopoly over a billion-euro teeth whitening industry. More pertinently, the re-interpretation of the *Act* to include teeth whitening, and changes to UK and European laws to allow teeth whitening markets to expand demonstrates how commercial power and scientific authority can reshape political systems.

These regulatory changes shape social understandings of oral health. Medicalisation of tooth colour reframes ageing and natural variation as pathological problems requiring intervention, positioning white teeth as an aesthetic ideal shaped through commercialised norms. Illich [54] described such dynamics as social iatrogenesis, the societal harms caused by medical institutions fostering dependency on health services which curtails personal freedoms [54]. Our findings further highlight how broader democratic autonomy and freedoms are subordinated when medical and scientific authority is mobilised within commercial and political processes. Such tensions are reflected in legislation that regulates who can touch or speak of an aspect of the body (mouth), particularly in relation to teeth whitening products deemed safe for public use.

**Figure 4:**
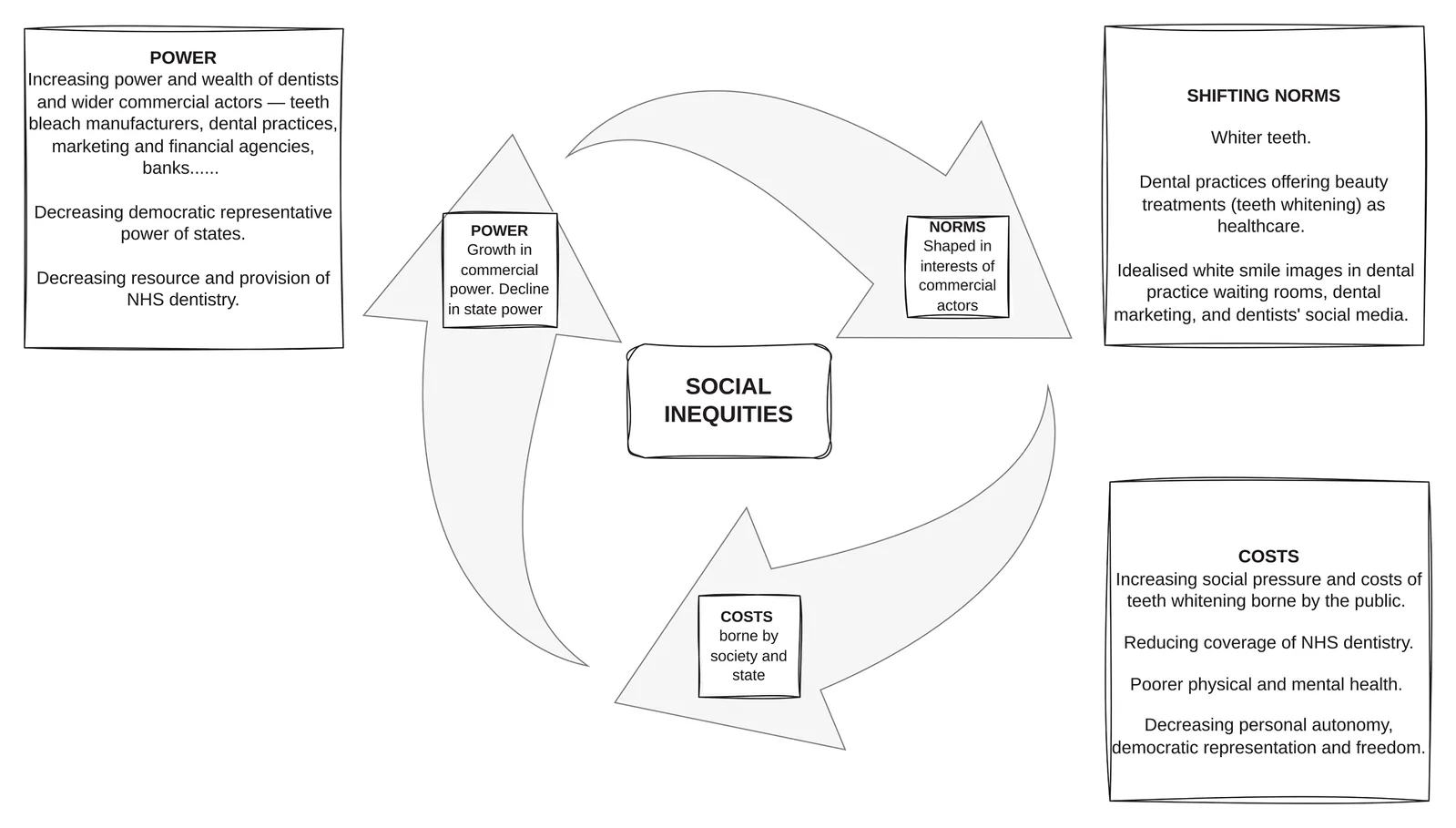
Commercial actors increasing their power and reproducing historically entrenched social inequities.

The IE feminist lens surfaces power imbalances in these political processes. Democratic institutions such as the European Parliament, Courts and the GDC (who are accountable to the British Parliament) privilege institutional constructions of safety and limit scrutiny of dentists’ commercial interests and power. The courts deference to dentists’ scientific authority may have undermined a neutral evaluation of Jamous’ claims. Moreover, medical actors’ scientific authority increases their own wealth and power, while also reshaping political systems in ways that reinforce wider global corporate power. This demonstrates biomedicalisation, the complex, multisited and multidirectional forms of medicalisation that encompasses not only dentists and medicine but global capitalist dynamics, including corporates, democratic and financial institutions [55] (Figure 4).

Teeth whitening is commonly positioned as a gateway procedure for upselling more extensive cosmetic dentistry [56, 57] with some patients reporting debts exceeding £25 000 [58]. Debt therefore becomes an ongoing mechanism through which capital value extraction continues beyond the point of treatment, extending processes of capital accumulation into long-term financial obligations. In parallel, dental practices increasingly act as credit brokers by offering finance plans for such treatments which normalises idealised white smiles and treatment-related debt. Until 1997, the GDC banned dental practices from promotional marketing, but these restrictions have relaxed [57, 59]. Idealised body images (found in dental practices and dentists’ social media) and financial debt are known to influence mental health [60, 61].

The financialisation of cosmetic dentistry is evident as expansion of markets. Globally, in 2022, the cosmetic dentistry market was estimated at $33.6 billion and projected to reach $89.03 billion by 2030. In the UK, it is projected to increase from $1.7 billion to $4.2 billion by 2030, and therefore surpassing the full UK government budget for NHS dentistry which sits at £3 billion [7, 62, 63]. This expansion can further narrow beauty ideals and create body image concerns which disproportionately impact women, minoritised communities and those living with facial difference, for whom appearance comes under heavier scrutiny [64, 65]. As cosmetic dental markets expand, concerns also arise about the redirection of dental resources. Currently, the provision of state-funded NHS dentistry is at a historic low. A 2024 UK analysis showed that 96.9% of new patients attempting NHS dentistry access were unsuccessful. Of those, 11% sought private treatments [66] which is less affordable for marginalised populations who often experience a greater burden of dental diseases [66–69]. From a feminist and Black radical perspective, these dynamics reflect how the commercial determinants of health are embedded in patriarchal, racialised and ableist power relations through which markets such as teeth whitening reshape social norms of appearance and access to dental healthcare [27, 70] to reproduce and reinforce historically entrenched social inequities (Figure 4).

In conclusion, by examining how power was exercised to expand the practice of teeth whitening, this study illustrates the operation of the commercial and political determinants of health in dentistry. It shows there is no objective scientific boundary between beauty and healthcare. Instead, boundaries are negotiated and reshaped through intersecting forms of professional, scientific, commercial and political power operating through texts. Texts (the Dentists Act 1984, SOP, Cosmetics Directive…) are not passive documents; they are mobilised by institutions such as the European Council of Dentists and the GDC to position teeth whitening by beauticians as a crime and the practice of dentistry. These findings raise warnings for the future. The SOP is an evolving document to which new cosmetic procedures are added. Since GDC v. Jamous, “non-surgical cosmetic injectables” (Botox and filler injections) have been included in an updated SOP [71]. This shows that texts, and therefore, ruling relations are not static; they are re-read and re-interpreted by those with capital and power. Power (e.g., commercial and scientific) can draw in additional power (e.g., professional and political) and amplify its reach. Further research is needed to examine how intersecting systems of power, including patriarchy and racism, shape experiences of dentistry, and to understand how commercial conflicts of interest embedded within institutional systems can be recognised and mitigated within health services.

## Figures and Tables

**Table 1:** Local data: Ethnographic observations and interviews.

Ethnographic observations	In-depth interviews with dentists *(n* = 8)	In-depth interviews with patients/public (*n* = 16)	Diary interviews patients/ public (*n* = 3)
British Dental Conference and Dentistry Shows 2018 and 2019—co-hosted by the BDA (4 days)—approx. 10 000 delegates, 400 exhibitors. Attended seminars delivered at the BDA, British Academy Aesthetic and Cosmetic Dentistry (BACD) and the Digital Dentist Theatre and the Facial Aesthetics Theatre. Cosmetic dentistry treatment sessions: Observed 9 patients’ treatments (M:F = 3:6) 5 settings (4 dental practices and 1 quasi-clinical setting in a cosmetic dentist’s home) 15 appointments observed (treatments observed: teeth whitening, veneers, Botox and filler injections) Contextual interviews of five dentists whilst at the field sites (M:F = 2:3) Cosmetic dentistry training (two 4-h sessions) delivered to dentists	M:F = 5:3 White: BME = 5:3	M:F = 2:14 White: BME = 14:2 Patients: Public = 12:4	M:F = 1:2 White: BME = 3:0 Patients: Public = 2:1 Diaries written/ recorded on 3 consecutive days on WhatsApp about social pressures to have cosmetic dentistry. Diaries included images such as teeth whitening adverts

*Note:* In-depth interviews were audio-recorded and transcribed verbatim.

**Table 2:** Recurring discourse of crime.

Ethnographic observations	Direct quotes from dentists	Institutional texts
Talk from the fieldwork describing beauticians as: Charlatans Not medically trained Unsafe Should be shopped^a^ R.L. and B.J.G. had conversations with the policy staff and members of the GDC who said ‘any hand to mouth action’ is dentistry including any form of teeth whitening.	“...I know there’s some illegal stuff going on, with like beauty salons and whitening.” (Safwan 2019) “Well, they’re breaking the law [beauticians] so they shouldn’t be doing it...” (Safwan 2019) “Well it’s illegal for a start. I mean tooth whitening by beauticians is illegal...”(Richard 2018) “I was like it doesn’t matter that it’s not the same bleach you [non-dentists] still can’t be bleaching patients” (Francesca, 2018) “according to the GDC, if you sit them there and tell them—breaking the law” (Francesca 2018)	“Offering or providing dental treatments [teeth whitening] when not registered with the GDC is illegal practice.” [GDC Tooth Whitening and Illegal Practice Webpage, 2018] “Products that contain or release less than 0.1% hydrogen peroxide can be legally sold in Europe but that does not mean that unregistered individuals using them to provide tooth whitening treatment makes that treatment legal or safe.” [General Dental Council, Standards and guidance, 2026] [31]

a ‘shopped’—to shop someone is UK English colloquial for reporting someone to the authorities, for example, to the police.

**Table 3:** The work of actors, and the texts they wielded, to introduce and resist higher peroxide concentration teeth whitening gel in the UK market [35–37].

Year	Work	Institutions/Actors	Type of actor(s)	Text wielded
1992	Opalescence (3.4% peroxide) launched in UK	Optident (bleach product distributor) Ultradent Inc. (bleach product manufacturer)	Commercial	EU Cosmetics Directive (breached)
	Opalescence’s sale was blocked by Department of Trade and Industry due to its peroxide content. Product withdrawn from the UK market	UK Department of Trade and Industry	Democratic (executive arm of the state that implements laws)	EU Cosmetics Directive
1994	Ultradent applied for a Medical Devices CE Mark for Opalescence. The German Notified Body RWTUV certified Opalescence with a CE mark. Therefore, the whitening gel could be distributed across the European Economic Area as a medical device	EU Notified Body	Commercial (EU notified bodies are private commercial organisations undertaking state functions of quality and safety assurance of medical devices)	EU Medical Devices Directive
1995	Opalescence rebranded and relaunched in the UK as a medical device	Optident and Ultradent	Commercial	EU Medical Devices Directive
	Opalescence’s sale blocked again by the Department of Trade and Industry because it still considered it a cosmetic product and not a medical device	UK Government Department of Trade and Industry and Department of Health	Democratic	EU Cosmetics Directive (breached)
1996	Ultradent and Optident sued two UK Government Departments (Department of Trade and Industry and Department of Health) for withdrawing a medical device. Opalescence was judged as a medical device by the High Court	Courts	Democratic (Judiciary branch of the state that interprets and apply laws)	EU Cosmetics Directive (by state actors — UK government departments) EU Medical Devices Directive (by commercial actors)
1999	Department of Trade and Industry and Department of Health appealed the High Court decision Appeal judges ruled that Opalescence is a cosmetic product not a medical device. Judgement based on the premise that teeth discolouration is not a condition, disease, physical and mental disability and judged it necessary to demarcate the fields of cosmetics and pharmaceuticals	Courts (Appeal judges)	Democratic	EU Cosmetics Directive
2001	Optident and Ultradent further appealed the decision in the House of Lords House of Lords ruled teeth whitening products are governed by the EU Cosmetics Directive and not the Medical Devices Directive	Parliament	Democratic	EU Medical Devices (by commercial actors) EU Cosmetics Directive (by Parliament)

**Table 4:** Actors, their divergent interests, and lines of accountability involved in expanding and restricting the UK and European teeth whitening markets.

Actor	Key functions/interests	Accountable to	Type of determinant of health
British Dental Association	Dentists’ trade union, therefore, works to secure dentists’ financial interests and labour rights	Members (dentists) who are both employed and employers	Commercial and political
Council of European Dentists—constitutes 33 dental organisations across 31 EU countries	Represents professional, clinical and regulatory interests of dentists. Some of the professional organisations such as BDA and Norwegian Dental Association have a trade union function	Members (dentists)	Commercial and Political
Courts	Ensuring democratically established laws are applied equally across actors including governments and large corporations	Judiciary is a key plank of democracy by protecting democratic laws equally and fairly. Indirectly accountable to the public.	Political
Department of Health	Responsible for public health, healthcare policy and the NHS	Led by democratically elected Secretary of State, therefore, accountable to democratic institutions—Parliament. Indirectly accountable to the public	Political
Department of Trade and Industry	Responsible for business trade, industry policy and consumer protections	Led by democratically elected Secretary of State, therefore, accountable to democratic institutions—Parliament. Indirectly accountable to the public	Commercial and Political
European Commission	Executive (operational) branch of the European Union. Functions include proposing legislations	Constitutes 27 Commissioners. Each EU member state appoints (does not elect) one Commissioner, and together they form the College of Commissioners, which acts as the political leadership of the EU’s executive branch. Indirectly accountable to the public (EU citizens) via the European Parliament	Political
EU Notified Body	Tests, inspects and certifies products including medical devices for quality and safety. Currently there are 46 EU Notified Bodies for Medical Devices	In the UK, medical devices Notified Bodies are accountable to Medicines and Healthcare products Regulatory Agency (MHRA)—an executive agency of the Department of Health. Therefore, indirectly accountable to the public. But Notified Bodies are private for-profit commercial bodies, therefore, are commercial actors	Commercial and Political
General Dental Council	Core function—protect patient safety and maintain public confidence in the dental profession	Professional Standards Authority (PSA) that is accountable to Parliament. Directly and indirectly accountable to the public	Political
House of Lords	Upper chamber of UK Parliament—conducts detailed scrutiny of legislations	Not directly accountable to anybody. It is constrained by the House of Commons (the principal democratically elected lower chamber of UK Parliament) through constitutional limits. Indirectly accountable to the public	Political
Optident	UK based supplier and distributor of dental products	Shareholders (commercial actors)	Commercial
Ultradent	American dental manufacturing company headquartered in South Jordan, Utah with global reach. Products sold across 130 countries	Shareholders (commercial actors)	Commercial

## Data Availability

The data that support the findings of this study are available on request from the corresponding author. The data are not publicly available due to privacy or ethical restrictions.

## References

[1] Carey CM (2014). Tooth Whitening: What We Now Know. Journal of Evidence-Based Dental Practice.

[2] Eachempati P, Kumbargere Nagraj S, Krishanappa S Kiran Kumar, Gupta P, Yaylali IE (2018). Home-Based Chemically-Induced Whitening (Bleaching) of Teeth in Adults. Cochrane Database of Systematic Reviews.

[3] Mintel Consulting (2006). British Academy of Cosmetic Dentistry Survey. London.

[4] Dental Council of New Zealand (2009). Practice Statement for Cosmetic Dentistry. http://www.dcnz.org.nz/assets/Uploads/Codes-of-practice/Practice-Statement-for-Cosmetic-Dentistry-Final.pdf.

[5] Alkhatib MN, Holt R, Bedi R (2004). Prevalence of Self-Assessed Tooth Discolouration in the United Kingdom. Journal of Dentistry.

[6] Grand View Research Teeth Whitening Market (2024–2030) [Market Analysis Report GVR-4-68039-944-7]. https://www.grandviewresearch.com/industry-analysis/teeth-whitening-market-report.

[7] Grand View Research (2025). UK Cosmetic Dentistry Market Size & Outlook, 2022–2030. https://www.grandviewresearch.com/horizon/outlook/cosmetic-dentistry-market/uk#:~:text=UK%20cosmetic%20dentistry%20market%20highlights,cosmetic%20dentistry%20market%20in%202022.

[8] Fortune Business Insights (2026). Teeth Whitening Market Size, Share & Industry Analysis, by Product (Whitening Toothpaste, Whitening Gels & Strips, and Light Teeth Whitening Devices), by Distribution Channel (Supermarkets/Hypermarkets, Pharmacy Stores, Online, and Others), and Regional Forecast, 2026–2034. https://www.fortunebusinessinsights.com/teeth-whitening-market-110349.

[9] Gilmore AB, Fabbri A, Baum F (2023). Defining and Conceptualising the Commercial Determinants of Health. Lancet.

[10] Kickbusch I (2015). The Political Determinants of Health-10 Years on. BMJ.

[11] Peres MA, Macpherson LMD, Weyant RJ (2019). Oral Diseases: A Global Public Health Challenge. Lancet.

[12] Holden ACL, Adam L, Thomson WM (2022). Dentists’ Perspectives on Commercial Practices in Private Dentistry. JDR Clinical & Translational Research.

[13] Spillane B, Gallagher J, Witton R, Patel R (2025). The Changing Market of Dentistry in England. Faculty Dental Journal.

[14] European Parliament (2009). Regulation EC No 1223/2009 of the European Parliament and of the Council of 30th November 2009 on Cosmetic Products (Recast). https://eur-lex.europa.eu/legal-content/EN/TXT/?uri=CELEX%3A02009R1223-20160812.

[15] The Cosmetic Products Enforcement Regulations 2013.

[16] EU Directive (2011). COUNCIL DIRECTIVE 2011/84/EU of 20 September 2011 Amending Directive 76/768/EEC, Concerning Cosmetic Products, for the Purpose of Adapting Annex III Thereto to Technical Progress. http://eur-lex.europa.eu/legal-content/EN/TXT/?uri=uriserv:OJ.L_.2011.283.01.0036.01.ENG.

[17] Holden ACL (2018). A Whiter Shade of Grey: Comparisons of the Legal and Professional Status of Tooth Whitening in Three Jurisdictions. Journal of Law and Medicine.

[18] Lala R, Jamieson LM, Saxena S (2026). Overcommercialisation of Dental Practice in England and Decline in Oral Health. BMJ.

[19] Dental Nursing (2025). https://www.dental-nursing.co.uk/content/news/don-t-say-cheese-two-thirds-of-adults-are-too-self-conscious-to-smile-in-photos.

[20] Nettleton S (1992). Power, Pain and Dentistry.

[21] Smith DE (2005). Institutional Ethnography: A Sociology for People.

[22] Rankin J (2017). Conducting Analysis in Institutional Ethnography: Guidance and Cautions. International Journal of Qualitative Methods.

[23] Rankin J (2017). Conducting Analysis in Institutional Ethnography: Analytical Work Prior to Commencing Data Collection. International Journal of Qualitative Methods.

[24] Collins P Hill (2000). Black Feminist Thought: Knowledge, Consciousness, and the Politics of Empowerment.

[25] Bourdieu P (1986). Distinction: A Social Critique of the Judgement of Taste.

[26] Smith DE (2006). Institutional Ethnography as Practice.

[27] James S (1972). International Wages for Housework Campaign (IWFHC).

[28] Smith DE, Turner SM (2014). Incorporating Texts Into Institutional Ethnographies.

[29] Geertz C, Darnton R (2017). The Interpretation of Cultures.

[30] O’Brien BC, Harris IB, Beckman TJ, Reed DA, Cook DA (2014). Standards for Reporting Qualitative Research: A Synthesis of Recommendations. Academic Medicine: Journal of the Association of American Medical Colleges.

[31] General Dental Council (2026). Working Within the Law [Standards and Guidance]. https://www.gdc-uk.org/standards-guidance/standards-and-guidance/working-within-the-law/what-is-the-legal-position.

[32] British Dental Association (2019). Teeth Whitening. https://bda.org/about-the-bda/campaigns/teethwhitening.

[33] Dental Protection (2014). Dental Protection. https://www.dentalprotection.org/uk/articles/tooth-whitening.

[34] DBIS (2012). Consumer Protection. The Cosmetic Products (Safety) (Ammendment) Regulations.

[35] House of Lords (2001). Judgments—Optident Limited and Another v Secretary of State for Trade and Industry and Another. [2001] UKHL 32. https://publications.parliament.uk/pa/ld200102/ldjudgmt/jd010628/optid-1.htm.

[36] Kelleher M, Greenwall L (2001). Bleaching Techniques in Restorative Dentistry: An Illustrated Guide.

[37] Morris CDN (2003). Tooth Whiteners—The Legal Position. British Dental Journal.

[38] Great Britain (1984). The Dentists Act 1984 c24.

[39] General Dental Council (2019). General Dental Council Information, Standards & Guidance. https://www.gdc-uk.org/information-standards-guidance/information-on-dental-treatment/tooth-whitening-and-illegal-practice/what-is-the-legal-position.

[40] Harris S (2013). Case Update: General Dental Council v Jamous, [2013] EWHC 1428. https://www.kingsleynapley.co.uk/insights/blogs/regulatory-blog/case-update-general-dental-council-v-jamous-2013-ewhc-1428.

[41] BAILII (2013). General Dental Council v Jamous [2013] EWHC 1428.

[42] General Dental Council (2009). Scope of Practice. https://heeoe.hee.nhs.uk/sites/default/files/docustore/1279809682_ywJv_scope_of_practice_april_2009.pdf.

[43] Evans S (2015). Illegal Tooth Whitener Can’t Apply for a Judicial Review. https://dentistry.co.uk/2015/03/09/illegal-tooth-whitener-cant-apply-judicial-review.

[44] General Dental Council (2016). GDC Position Statement on Tooth Whitening.

[45] General Dental Council (2017). GDC Illegal Practice Prosecution Policy.

[46] (1987). Consumer Protection Act 1987. https://www.legislation.gov.uk/ukpga/1987/43/contents.

[47] General Dental Council (2013). Focus on Standards—The 9 Principles. https://standards.gdc-uk.org.

[48] Pleatsikas C (2015). Report From North America.

[49] O’Donoghue D, O’Neill L (2025). Illegal Teeth-Whitening Industry Exposed by BBC. https://www.bbc.co.uk/news/articles/c20z7xx6nr4o.

[50] Serban ST, Perdomo S, Borges-Yañez A (2025). The Political Determinants of Oral Health Inequalities: Lessons in Policy Development and Implementation From Six Case Studies. Community Dentistry and Oral Epidemiology.

[51] Bourdieu P, Dirks NB, Eley G, Ortner SB (1993). Culture/Power/History: A Reader in Contemporary Social Theory.

[52] Kickbusch I (2012). Addressing the Interface of the Political and Commercial Determinants of Health. Health Promotion International.

[53] Schmid HJ (2000). English Abstract Nouns as Conceptual Shells: From Corpus to Cognition.

[54] Illich I (1976). Limits to Medicine: Medical Nemesis: The Expropriation of Health.

[55] Clarke AE, Shim JK, Mamo L, Fosket JR, Fishman JR (2003). Biomedicalization: Technoscientific Transformations of Health, Illness, and U.S. Biomedicine. American Sociological Review.

[56] Dentistry (2024). Tooth Whitening as an Entry Point to Better Oral Health.

[57] Lala R (2020). The Great British Smile: An Institutional Ethnography of Power in Cosmetic Dentistry.

[58] Dentistry (2023). One Third of Under 35s Had Cosmetic Dental Treatment in the Last 12 Months.

[59] General Dental Council (1997). Maintaining Standards.

[60] Royal College of Psychiatrists (2017). Debt and Mental Health.

[61] Merino M, Tornero-Aguilera JF, Rubio-Zarapuz A, Villanueva-Tobaldo CV, Martín-Rodríguez A, Clemente-Suárez VJ (2024). Body Perceptions and Psychological Well-Being: A Review of the Impact of Social Media and Physical Measurements on Self-Esteem and Mental Health With a Focus on Body Image Satisfaction and Its Relationship With Cultural and Gender Factors. Health.

[62] Grand View Research (2025). Cosmetic Dentistry Market Size, Share & Trends Analysis Report by Product (Dental Systems & Equipment, Dental Implants, Dental Crowns & Bridges), by Region (North America, Europe), and Segment Forecasts, 2023–2030.

[63] Garratt K (2024). House of Commons Library. https://dentistry.co.uk/app/uploads/2024/06/CBP-9597.pdf.

[64] Picard A (2009). Making the American Mouth.

[65] Bordo S (2003). Unbearable Weight: Feminism, Western Culture, and the Body.

[66] Office for National Statistics (2025). Experiences of NHS Healthcare Services in England.

[67] Healthwatch (2021). Twin Crisis of Access and Affordability Calls for a Radical Rethink of NHS Dentistry.

[68] Office for Health Improvement and Disparities (2023). Official Statistics National Dental Epidemiology Programme (NDEP) for England: Oral Health Survey of 5 Year Old Children 2022.

[69] Office for Health Improvement and Disparities (2024). Adult Oral Health Survey 2021. https://www.gov.uk/government/statistics/adult-oral-health-survey-2021/adult-oral-health-survey-2021-report-summary.

[70] Robinson CJ (2021). Black Marxism: The Making of the Black Radical Tradition.

[71] General Dental Council (2013). Scope of Practice.

